# Effect of D-Glucuronic Acid and N-acetyl-D-Glucosamine Treatment during In Vitro Maturation on Embryonic Development after Parthenogenesis and Somatic Cell Nuclear Transfer in Pigs

**DOI:** 10.3390/ani11041034

**Published:** 2021-04-06

**Authors:** Joohyeong Lee, Eunhye Kim, Seon-Ung Hwang, Lian Cai, Mirae Kim, Hyerin Choi, Dongjin Oh, Eunsong Lee, Sang-Hwan Hyun

**Affiliations:** 1 Laboratory of Veterinary Embryology and Bio-technology (VETEMBIO), Veterinary Medical Center and College of Veterinary Medicine, Chungbuk National University, Cheongju 28644, Korea; durubit@gmail.com (J.L.); iwsleh@nate.com (E.K.); ghkdsun@hanmail.net (S.-U.H.); cailian005@nate.com (L.C.); kmr9309@naver.com (M.K.); hyrin3642@naver.com (H.C.); rosecafes123@naver.com (D.O.); 2 Institute of Stem Cell & Regenerative Medicine (ISCRM), Chungbuk National University, Cheongju 28644, Korea; 3 Graduate School of Veterinary Biosecurity and Protection, Chungbuk National University, Cheongju 28644, Korea; 4 College of Veterinary Medicine, Kangwon National University, Chuncheon 24341, Korea

**Keywords:** hyaluronic acid, glucuronic acid, N-acetyl-D-glucosamine, oocyte maturation, pig

## Abstract

**Simple Summary:**

Hyaluronic acid, also known as hyaluronan, is essential for the expansion of cumulus cells, the maturation of oocytes, and further embryo development. This study aimed to examine the effects of treatment with glucuronic acid and N-acetyl-D-glucosamine, which are components of hyaluronic acid, during porcine oocyte in vitro maturation and embryonic development after parthenogenetic activation and somatic cell nuclear transfer. We measured the diameter of mature oocytes, the thickness of the perivitelline space, the intracellular reactive oxygen species level, and the expression of cumulus cell expansion genes and reactive oxygen species-related genes and examined the cortical granule reaction of oocytes after electrical activation. In conclusion, the addition of 0.05 mM glucuronic acid and 0.05 mM N-acetyl-D-glucosamine and during the initial 22 h of in vitro maturation in pig oocytes has beneficial effects on cumulus expansion, perivitelline space thickness, cytoplasmic maturation, reactive oxygen species level, cortical granule exocytosis, and early embryonic development after parthenogenesis and somatic cell nuclear transfer. Glucuronic acid and N-acetyl-D-glucosamine can be applied to in vitro production technology and can be used as ingredients to produce high-quality porcine blastocysts.

**Abstract:**

This study aimed to examine the effects of treatment with glucuronic acid (GA) and N-acetyl-D-glucosamine (AG), which are components of hyaluronic acid (HA), during porcine oocyte in vitro maturation (IVM). We measured the diameter of the oocyte, the thickness of the perivitelline space (PVS), the reactive oxygen species (ROS) level, and the expression of cumulus cell expansion and ROS-related genes and examined the cortical granule (CG) reaction of oocytes. The addition of 0.05 mM GA and 0.05 mM AG during the first 22 h of oocyte IVM significantly increased oocyte diameter and PVS size compared with the control (non-treatment). The addition of GA and AG reduced the intra-oocyte ROS content and improved the CG of the oocyte. GA and AG treatment increased the expression of *CD44* and *CX43* in cumulus cells and *PRDX1* and *TXN2* in oocytes. In both the chemically defined and the complex medium (Medium-199 + porcine follicular fluid), oocytes derived from the GA and AG treatments presented significantly higher blastocyst rates than the control after parthenogenesis (PA) and somatic cell nuclear transfer (SCNT). In conclusion, the addition of GA and AG during IVM in pig oocytes has beneficial effects on oocyte IVM and early embryonic development after PA and SCNT.

## 1. Introduction

The high reproductive potential and low maintenance costs of pigs make this species a widely accepted animal model for the experiment. In addition, pigs are considered an ideal animal model for the study and development of new therapies for human diseases because of their genetic, anatomical, and physiological similarities to humans [1,2]. However, the in vitro production efficiency of porcine embryos is still low, which hinders its use as a model for research [3].

Oocyte quality is an important factor that influences early embryonic viability, establishment, and maintenance of pregnancy, fetal development [4]. The incomplete maturation of an oocyte through in vitro maturation (IVM) is one of the main factors that reduces embryo quality. Oocyte maturation can be divided into nuclear and cytoplasmic maturation. Although many studies have reported high rates (>90%) of nuclear maturation of oocytes in vitro [5,6], more studies are needed to improve the cytoplasmic maturation of oocytes in vitro. The cytoplasmic maturation of oocytes is greatly influenced by their interaction with cumulus cells; these cells play an important role in oocyte growth and maturation. They supply nutrients [7,8] and/or messenger molecules for the development of the oocyte [9,10] and mediate the effects of hormones on cumulus-oocyte complexes (COCs) [11]. Cumulus cell expansion occurs during oocyte maturation following the luteinizing hormone (LH) surge in vivo or following follicle-stimulating hormone (FSH) or epidermal growth factor (EGF) treatment in vitro. Cumulus expansion requires the synthesis and organization of the extracellular matrix (ECM), where hyaluronic acid (HA) is the main component [12].

HA, also known as hyaluronan or hyaluronate, is a straight-chain glycosaminoglycan polymer composed of repeating units of D-glucuronic acid (GA) and N-acetyl-d-glucosamine (AG) that is found in vertebrates and certain microorganisms. The biological functions of HA include maintaining the elasticity of liquid connective tissue, regulating tissue hydration, supramolecular assembly of proteoglycans in the ECM, cell adhesion, mitosis, migration, tumorigenesis, and numerous receptor-mediated functions related to wound healing and inflammation [13]. Particularly, HA is abundant in the reproductive tract and is essential for the cumulus cell expansion, as well as for the maturation of oocytes and further embryo development. HA is found in the ECM of animal reproductive organs and in the uterus, oviduct, and follicular fluids in mice, pigs, cattle, and humans [14]. In previous studies, the physiological concentration of HA in pig body fluids was found to be 0.04~1.83 mg/mL [15]. It is not clear how exogenous HA plays a role in oocyte maturation and embryonic development. HA is a major component of COCs and enhances the interaction between cellular receptors and molecules in the external environment [16]. It can be assumed that the interaction of the appropriate concentration of HA with the HA receptor (*CD44*) can positively affect oocyte maturation and embryonic development after fertilization. Furthermore, the supplement of HA into the oocyte or embryo culture medium improved the developmental competence of bovine oocytes and embryos and their quality [17,18]. The advantages of adding GA and AG to the culture medium are that they are not lipophilic or as large as HA (approximately 1000 kDa) and can therefore be easily incorporated into maturation media and maturing oocytes [19].

In general, porcine oocytes are cultured in two stages for IVM, and Gonadotropins are added during the first half. In addition, it has been reported that during the first half of IVM, the gap junction communication through cumulus cells to oocyte is actively progressing [20].

We hypothesized that when the components of HA were added to the IVM medium, the quality of porcine oocytes and embryo developmental competence after parthenogenetic activation (PA) and somatic cell nuclear transfer (SCNT) could be improved. To test this hypothesis, we measured the diameter of mature oocytes, the thickness of the perivitelline space (PVS), the intracellular reactive oxygen species (ROS) level, cumulus expansion, and the expression of cumulus cell expansion genes and ROS-related genes after IVM and examined the cortical granule (CG) reaction of oocytes after electrical activation.

## 2. Materials and Methods

### 2.1. Culture Media

Unless stated, all chemicals were purchased from Sigma-Aldrich Corporation (St. Louis, MO, USA). For IVM of immature oocytes, Medium-199 (M199; Invitrogen, Grand Island, NY, USA) to which 0.6 mM cysteine, 0.91 mM pyruvate, 1 µg/mL insulin, and 10 ng/mL EGF were added. We also added 0.1% (*w/v*) polyvinyl alcohol (PVA) for the defined medium or 10% (*v/v*) porcine follicular fluid (PFF) for the complex medium according to the experimental design. Porcine zygote medium-3 (PZM-3) containing 0.3% (*w/v*) bovine serum albumin (BSA) [21] plus 2.77 mM myo-inositol, 0.34 mM tri-sodium citrate, and 10 µM β-mercaptoethanol was used as the in vitro culture medium for embryo development.

### 2.2. Oocyte Collection and IVM

The ovaries used in the experiment were collected from the ovaries of slaughtered prepubertal gilts in a local slaughterhouse. The collected ovaries were immersed in physiological saline at 34–37 °C and transported to the laboratory within 1 h. After washing the ovaries several times with sterilized physiological saline, follicular fluid was aspirated from follicles with a diameter of 3–8 mm. Only COCs were selected and washed in HEPES-buffered Tyrode’s medium (TLH) containing 0.05% (*w/v*) PVA (TLH-PVA). Depending on the experimental design, 50–60 COCs in a group were placed into each well of a 4-well multi-dish (Nunc, Roskilde, Denmark) containing 500 μL of defined or complex medium with 80 µg/mL FSH (Antrin R-10, Kyoritsu Seiyaku, Tokyo, Japan) and 10 IU/mL human chorionic gonadotropin (hCG) (Intervet International BV, Boxmeer, The Netherlands). COCs were cultured at 39 °C in a humidified atmosphere of 5% CO_2_ in the air. After 22 h of maturation culture, the COCs were washed three times in a fresh hormone-free IVM medium and then cultured in IVM medium without hormones for 22 h. During the IVM, COCs were treated with GA and AG or left untreated, according to the experimental design.

### 2.3. Experimental Design

We first investigated the optimal concentration of GA or AG in a defined medium (M199 + 0.1% PVA). In experiment 1, we investigated the effect of GA at various concentrations (0, 0.01, 0.05, and 0.1 mM) in a chemically defined medium during IVM on embryonic development after PA. In experiment 2, we investigated the effect of AG at various concentrations (0, 0.01, 0.05, and 0.1 mM) in a chemically defined medium during IVM on embryonic development after PA. In experiment 3, we investigated the addition time of the optimal concentrations of GA and AG investigated in experiments 1 and 2. Immature oocytes were treated for 0–22, 23–44, or 0–44 h in chemically defined medium with the combined treatment comprising 0.05 mM GA and 0.05 mM AG added during IVM (designated GAAG0-22, GAAG23-44, and GAAG0-44, respectively). In experiment 4, cumulus cell expansion, oocyte diameter, and size of PVS in oocytes at the end of maturation were evaluated. In experiment 5, we investigated the effect of GAAG0-22 treatment on intracellular ROS levels in oocyte and fluorescent intensities of CG in oocyte after PA. In experiment 6, we investigated the effect of GAAG0-22 treatment on the levels of cumulus cell expansion-related genes and ROS-related genes in cumulus cells or matured oocytes. In experiment 7 and 8, we determined the effect of GAAG0-22 treatment in the complex medium (M199 + 10% PFF) for subsequent embryonic development after PA (in experiment 7) and SCNT (in experiment 8).

### 2.4. Measurement of Cumulus Cell Expansion, Oocyte Diameter, and PVS Thickness

**Figure 1:**
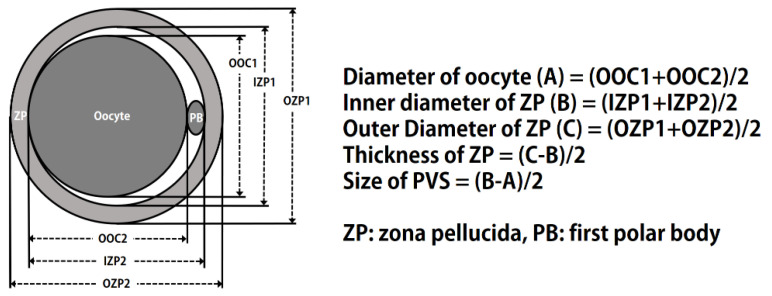
Diagram of the measurement of oocyte diameter, and the size of the perivitelline space (PVS) in porcine oocytes after in vitro maturation.

During IVM, images of COCs or denuded oocytes in each group were recorded at 200× magnification by using a digital camera (DS-L3; Nikon, Tokyo, Japan) attached to an inverted microscope (TE-300; Nikon). Cumulus cell expansion was performed as described previously [22], and confirmed by measuring each COC before and after maturation. To measure cumulus expansion, single COCs were matured in a 10 µL droplet of IVM medium covered with oil. Cumulus cell expansion during IVM was determined by the difference between the mean area of all COCs from each treatment at 0, 22, and 44 h. The size of each part of the matured oocytes was measured using image analysis software, and the size of the PVS was calculated as described previously [23] and Figure 1. COCs and oocytes in each group were measured using ImageJ software (version 1.46; National Institutes of Health, Bethesda, MD, USA).

### 2.5. Measurement of Intracellular ROS Activity

The level of ROS in oocyte cytoplasm was measured in 8–10 oocytes per treatment per replicate (three replicates). ROS were measured as previously procedures using 10 μM 2,7-dichlorodihydrofluorescein diacetate (H2DCFDA) to detect ROS as green fluorescence [24]. Oocytes from each treatment group were incubated for 20 min with an H2DCFDA drop. Following washing with PBS + 1% (*w/v*) BSA, the oocytes were immediately observed with an epifluorescence microscope (TE300; Nikon) equipped with UV filters (460 nm). The results were expressed as the relative fluorescence intensity from the ratio of fluorescence intensity of treated oocytes to that of non-treated oocytes.

### 2.6. PA

After in vitro maturation, only oocytes from which the first polar body was released were selected and placed between two wires of a 1 mm fusion chamber coated with an activation medium (280 mM mannitol, 0.05 mM MgCl_2_, 0.1 mM CaCl_2_). Oocyte activation was stimulated with a direct current (DC) pulse of 120 V/mm for 60 μs using Cell Fusion Generator (LF101; NepaGene, Chiba, Japan). Electrically stimulated oocytes were transferred to PZM-3 with 5 μg/mL cytochalasin B and cultured for 4 h at 39 °C temperature and in humidified atmosphere of 5% CO_2_, 5% O_2_, and 90% N_2_.

### 2.7. SCNT

SCNT was performed as previously described [25]. Donor cells (Fetal fibroblasts) were cultured in DMEM F-12 (Invitrogen) supplemented with 15% (*v/v*) FBS until a complete monolayer of cells was formed. The donor cells were synchronized at the G0/G1 stage of the cell cycle for 3–4 days by contact inhibition. A suspension of single cells was prepared by trypsinization using EDTA-trypsin and resuspending in TLH containing 0.4% (*w/v*) BSA (TLH-BSA) prior to cell injection. IVM oocytes were incubated for 10 min in TLH-BSA medium containing bisbenzimide H 33342, washed twice with fresh TLH-BSA medium, and then transferred to a droplet of TLH-BSA containing 5 μg/mL cytochalasin B covered with mineral oil. The first polar body and adjacent cytoplasm containing the chromosomes were enucleated aspiration using a 16 μm beveled glass pipette (Origio, Målov, Denmark). A donor cell was inserted into the PVS of an enucleated oocyte. The reconstructed single cell-oocyte couplets were fused by a DC pulse of 175 V/mm for 30 μs using an Electro Cell Fusion Generator in fusion medium consisting of 280 mM mannitol, 0.05 mM MgCl_2_, 0.1 mM CaCl_2_. After incubating in TLH-BSA for 1 h, oocytes with confirmed fusion were then activated in the same methods as PA. Electrically stimulated oocytes were transferred to PZM-3 supplemented with 1.9 mM 6-dimethylaminopurine combined with 0.4 μg/mL demecolcine for 4 h at 39 °C temperature and in a humidified atmosphere of 5% CO_2_, 5% O_2_, and 90% N_2_.

### 2.8. Embryo Culture

The PA and SCNT embryos were washed three times in fresh PZM-3, transferred into 30 μL droplets of PZM-3, and then cultured at 39 °C in a humidified atmosphere of 5% CO_2_, 5% O_2_, and 90% N_2_ for 7 days. Cleavage and blastocyst formation were evaluated on days 2 and 7, respectively, with the day of PA or SCNT was considered as Day 0. The total cell number of blastocysts was counted after staining with bisbenzimide H33342 under an epifluorescence microscope.

### 2.9. CG Determination

CG were visualized using fluorescent-labeling techniques, as previously described [26]. After PA, oocytes were fixed in 4% (*w/v*) paraformaldehyde in PBS for 1 h, then incubated in 0.5% (*w/v*) Triton X-100 in 0.1% (*w/v*) sodium citrate for 30 min, and stained with 5 mg/mL fluorescein isothiocyanate-labeled peanut agglutinin (FITC-PNA) for 30 min at 20–25 °C. Oocytes were then examined under an epifluorescence microscope equipped with UV filters (460 nm). The images were recorded digitally, and the fluorescence of oocytes stained with FITC-PNA was calculated using ImageJ software (version 1.46). Data were normalized to those obtained for untreated control PA embryos.

### 2.10. Gene Expression Analysis by Real-Time PCR

First, the expression levels of genes related to cumulus cell expansions (mRNA expression levels of *CD44* and *CX43*) were evaluated in cumulus cells from each treatment group. Second, the mRNA expression of ROS-related genes (*GSR*, *PRDX1*, *TXN2*, and *SOD1*) was evaluated in oocytes from each treatment group after IVM. To analyze gene expression levels in oocytes and cumulus cells after IVM, oocytes and cumulus cells were isolated from 60 COCs from each group by using 0.1% hyaluronidase. For the sampling of qRT-PCR using oocytes, 50–60 denuded MII oocytes were used. The oocytes and cumulus cells were washed twice in DPBS and stored at −70 °C for further analysis. Briefly, the total RNA was extracted from samples using RNAiso Plus (Takara Bio. Inc., Otsu, Shiga, Japan) according to the manufacturer’s instructions. Complementary DNA (cDNA) was prepared by subjecting 1 μg of the total RNA to reverse transcription using Reverse Transcription 5× Master Premix (ELPIS Bio. Inc., Deajeon, Korea). Gene expression was analyzed by real-time PCR (CFX96 Touch Deep Well Real-Time PCR Detection System; BIO-RAD, Hercules, CA, USA). After mRNA extraction and cDNA synthesis, qRT-PCR was performed using 2 μL of cDNA template with 10 μL 2× SYBR Premix Ex Taq (Takara Bio Inc., Shiga, Japan) containing primers specific to *CD44*, *CX43*, *GSR*, *PRDX1*, *TXN2*, and *SOD1* (Table 1). Reactions were performed for 40 cycles under the following conditions: denaturation at 95 °C for 30 s, annealing at 57 °C for 15 s, and extension at 72 °C for 30 s. Gene expression was quantified relative to the reference gene *GAPDH* (for cumulus) or *RN18S* (for oocytes). Relative quantification was based on a comparison of the threshold cycle (Ct) at a constant fluorescence intensity. Relative mRNA expression (R) was calculated using the following equation: R = 2^−[ΔCt sample−ΔCt control]^. Expression values were normalized to those of *GAPDH* or *RN18S*.

### 2.11. Statistical Analysis

The statistical analyses were conducted using Statistical Analysis System software (version 9.4; SAS Institute, Cary, NC, USA). The data were analyzed using a general linear model procedure followed by the least-significant-difference mean separation procedure when there were differences between treatments. Percentage data (e.g., rates of maturation, cleavage, blastocyst) were arcsine-transformed prior to analysis to maintain homogeneity of variance. The results are expressed as mean ±standard error of the mean (SEM). Probability values less than 0.05 were considered to be statistically significant unless otherwise stated.

## 3. Results

### 3.1. Effect of GA Concentration during IVM on Nuclear Maturation and Blastocyst Development after PA

We investigated the effect of GA treatment at different concentrations (0, 0.01, 0.05, and 0.1 mM) during IVM on the nuclear maturation and subsequent embryonic development of PA embryos. No significant difference was observed in the nuclear maturation between examined groups (91.3–94.7%). GA treatment during IVM did not alter PA embryonic development to cleavage (85.5–93.6%) and blastocyst (42.0–51.0%). However, the number of cells in blastocysts after PA was significantly higher in the 0.05 mM GA treatment group than in the untreated group (38.0 vs. 31.5 per blastocyst, *p* < 0.05) (Table 2).

### 3.2. Effect of AG Concentration during IVM on Nuclear Maturation and Blastocyst Development after PA

We investigated the effects of different concentrations of AG (0, 0.01, 0.05, and 0.1 mM) during IVM on the nuclear maturation and subsequent embryonic development of PA embryos. No significant difference in nuclear maturation was observed among the groups examined (95.0–97.2%). Cleavage rates were significantly higher in PA embryos treated with 0.05 mM AG compared with those of the untreated group (91.8% vs. 85.0%, *p* < 0.05). The rate of blastocyst formation was higher in the 0.05 mM AG-treated PA embryos compared with the untreated and 0.1 mM AG-treated embryos (59.6% vs. 46.3% and 45.2%, respectively, *p* < 0.05). Cell number in the blastocysts after PA was not influenced by AG treatment during IVM (40.0–43.1 per blastocyst) (Table 3).

### 3.3. Effect of Addition Time of GA and AG during IVM on Nuclear Maturation and Blastocyst Development

Immature oocytes were treated for 0–22 or 23–44 or 0–44 h in IVM medium with 0.05 mM GA and 0.05 mM AG (designated as GAAG0-22, GAAG23-44, and GAAG0-44, respectively). Oocytes cultured with GAAG0-22 presented increased blastocyst formation after PA compared with the control and GAAG23-44 (53.6% vs. 43.9–44.5%); however, the difference from GAAG0-44 (49.2%) was not significant. There was no significant difference in nuclear maturation (92.5–94.9%), cleavage rate (89.2–94.5%), or blastocyst cell number (39.0–41.0 per blastocyst) after PA (Table 4).

### 3.4. Effect of GA and AG Supplementation during IVM on Oocyte Cumulus Cell Expansion, Diameter, and Size of the PVS in Defined and Complex Medium

**Figure 2:**
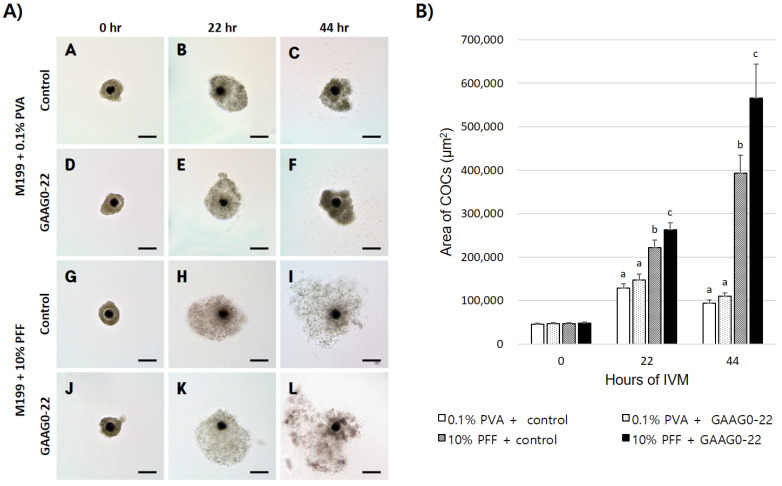
Evaluation of the cumulus expansion in a defined medium or complex medium. (**A**) Representative images of cumulus–oocyte complexes (COCs) taken at three time points (0, 22, and 44 h) during in vitro maturation. Scale bar, 200 μm. COCs were cultured in the defined medium (A–F), or 10% PFF medium (G–L); COCs were cultured without treatment (A–C, G–I) or cultured for 22 h in an IVM medium containing 0.05 mM GA and 0.05 mM AG (GAAG0-22) (D–F, J–L). (**B**) The area of each COCs was analyzed by drawing a line on the outer layer of the cumulus cell and measuring its inner area. The experiment was repeated three times with 25 oocytes per group. (^a, b, c^) Values with different superscripts within the same column are significantly different (*p* < 0.05).

When 0.05 mM GA and 0.05 mM AG were added to the IVM medium with 10% PFF during the initial 22 h of IVM, cumulus cell expansion was significantly increased at 22 and 44 h after maturation compared with the control or with IVM medium containing 0.1% PVA. After IVM, oocyte diameter was significantly greater (*p* < 0.05) in oocytes cultured with GAAG0-22 (111.5 μm) than in oocytes in the control group (110.0 μm). Furthermore, the PVS was significantly larger (*p* < 0.05) in oocytes cultured with GAAG0-22 (5.08 μm) than in oocytes in the control group (4.35 μm) and in those cultured with GAAG23-44 (4.40 μm) (Figure 2, Table 5).

### 3.5. Effect of GA and AG Supplementation during IVM on ROS Content of the Oocyte and Intensities of CG of PA Embryo

The intraoocyte ROS content was significantly lower in oocytes in the GA and AG treatment groups than in untreated oocytes (0.70–0.91 vs. 1.00 pixel per oocyte, *p* < 0.05). After PA, fluorescent intensities of CG were significantly lower (*p* < 0.05) in oocytes cultured with GAAG0-22 (0.9) than in oocytes in the control group (1.0) (Table 6).

### 3.6. Effect of GA and AG Supplementation during IVM on Gene Expression in Oocytes and Cumulus Cells

**Figure 3:**
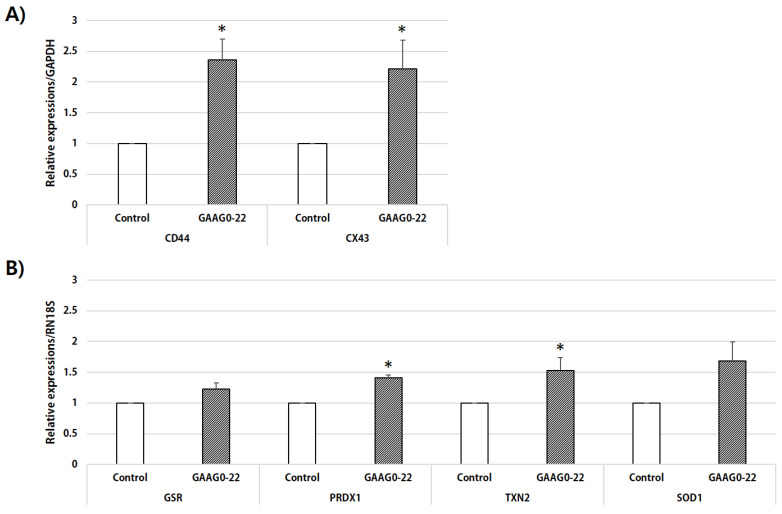
The mRNA expression levels of cumulus expansion- and antioxidant-related genes. (**A**) mRNA expression levels of genes related to cumulus cell expansion (*CD44*, *CX43*) were evaluated in cumulus cells from each treatment group, and (**B**) mRNA expression levels of ROS-related genes (*GSR*, *PRDX1*, *TXN2*, and *SOD1*) were evaluated in oocytes from each treatment group after in vitro maturation. (*) Values with different superscripts within the same column are significantly different (*p* < 0.05); four replicates.

To evaluate the effects of GA and AG on the expression of genes related to cumulus cell expansion, we performed real-time PCR to analyze the mRNA levels of *CD44* and *CX43* in cumulus cells (Figure 3A). *CD44* and *CX43* gene expression was significantly increased in cumulus cells treated with GA and AG compared with those in the control groups (*p* < 0.05). To evaluate the effects of GA and AG on the expression of ROS-related genes, we analyzed the mRNA expression levels of *GSR*, *PRDX1*, *TXN2*, and *SOD* in MII oocytes (Figure 3B). Gene expression of *PRDX1* and *TXN2* was significantly higher in oocytes treated with GA and AG than in those from the control group (*p* < 0.05).

### 3.7. Effect of GA and AG Supplementation in the Complex Medium during IVM on Embryonic Development after PA

In both the complex (M199 + 10% PFF) and chemically defined medium (M199 + 0.1% PVA), the blastocyst rates of oocytes derived from GAAG0-22 with PFF were significantly higher (*p* < 0.05) than those of the control and GAAG0-22 with PVA groups (66.6% vs. 52.8–53.0%) after PA. The cell number of blastocyst derived from complex medium were significantly higher (*p* < 0.05) than those for GAAG0-22 with PVA (43.9–45.4% vs. 37.5%) after PA. There was no significant difference in the cleavage rate (89.7–92.7%) (Table 7).

### 3.8. Effect of GA and AG Supplementation during IVM on Embryonic Development after SCNT

The SCNT embryos derived from GAAG0-22 oocytes exhibited a significantly higher (*p* < 0.05) rate of blastocyst formation than the control (25.1% vs. 16.0%). However, there was no significant difference in cleavage rate (80.9–84.7%) and blastocyst cell number (38.7–41.8 per blastocyst) after SCNT (Table 8).

## 4. Discussion

During the expansion of cumulus cells, changes in metabolic activity and morphology of cumulus cells affect the maturation of oocytes and their ability to develop after fertilization [27]. Fertilization of mammalian oocytes requires interactions between spermatozoa and the expanded cumulus ECM that surrounds the oocyte. This study aimed to examine the effects of treatment with components of HA, during porcine oocyte IVM. Cumulus expansion is based on ECM synthesis by cumulus cells. HA is the most abundant component of this ECM, and GA and AG are heterodimeric components of HA.

In this study, we first determined the optimal concentration and timing of supplementing the defined medium with GA and AG. The results demonstrated that supplementing the medium with 0.05 mM GA and 0.05 mM AG improved the developmental competence of PA embryos. In previous studies, it has already been reported that combination treatment of GA and AG is effective in the development of in vitro fertilized embryos [19,28]. In this study, when GA and AG were individually treated with a defined medium, they had a positive effect on embryonic development, so a combination treatment was selected. Moreover, these components of HA were added to the culture medium during the initial 22 h of IVM, and a higher percentage of PA embryos reached the blastocyst stage. These results are related to gap-junctional communication (GJC), which plays a central role in oocyte maturation. COCs cultured in vitro undergo GJC action during the initial 24 to 32 h of IVM [20], during this period, beneficial factors related to cytoplasmic maturation can migrate from cumulus cells to oocytes. So, it can be speculated that adding GA and AG during the first 22 h of the IVM would have had a positive effect on oocyte maturation.

Interestingly, in our study, supplementing a defined medium with GA and AG did not affect the expansion of cumulus cells, but had a positive effect on the diameter of the oocyte and the size of PVS, and reduced ROS in the oocyte. PVS enlargement can be explained by HA synthesis by oocytes and the binding of water molecules by HA [29]. Moreover, regardless of cumulus cells, the PVA enlargement can be induced by the production of HA by the oocytes. [29]. Our results also suggest that the addition of GA and AG directly affected oocytes, regardless of the cumulus expansion of oocytes. Previous studies showed that oocyte diameter [30] and PVS size [23] can be used as indicators to assess the quality of oocytes. PVS size is closely related to meiotic competence, chromatin configuration, and embryonic development in humans [31], mice [32], and pigs [33]. In addition, previous studies have demonstrated that supplementation with GA increases PVS in porcine oocytes [19]. According to the results of Current and Whitaker’s study [28], supplementation with 0.01 mM GA and 0.01 mM AG has beneficial effects on reducing polyspermy by increasing PVS size and improving early embryo development in pigs. Although the concentration and timing of the addition of GA and AG were different in our study, the results showing an increased size of the PVS are consistent. The concentrations of GA and AG required in the IVM process for porcine oocytes might vary depending on the characteristics of the culture medium. In contrast to the defined medium, in the culture to which PFF has been added, the required amount can be reduced. This is because the follicular fluid contains the component of HA [34]. Because oocytes and expanded cumulus cells contribute to increasing the amount of HA in PVS [35], supplementation of GA and AG during oocyte maturation may improve factors associated with PVS. Our results support previous findings that porcine oocytes with a large PVS through the supplementation of GA and AG exhibit greater cytoplasmic maturation and developmental competence.

In our study, ROS levels were lower in oocytes that matured in IVM medium supplemented with GA and AG than in oocytes in the control group. Moreover, RT-PCR analysis of ROS-related genes revealed that the expression of *PRDX1* and *TXN2* in GA- and AG-treated oocytes were significantly increased and that the expression of *GSR* and *SOD1* also tended to increase compared with oocytes in the control group (*p* < 0.07). Culture media containing HA can alter glutathione synthesis and expression of other antioxidant genes through HA-*CD44* receptor interactions [36]. Our data may suggest that the addition of GA and AG increased the expression of *CD44* in cumulus cells and that oocytes with high levels of *CD44* expression presented improved glutathione synthesis and ROS defense.

The results of this study indicated that CG exocytosis was more complete in oocytes matured with GA and AG supplementation compare with non-treated oocytes. CG is widely used as a criterion for evaluating the maturity and organelle tissue of developing oocytes, and CG migration is an important step in cytoplasmic maturation [37]. Mammalian CG first appears during the early stages of oocyte growth. The CG of the fertilized oocyte undergoes exocytosis, releasing its contents into PVS. After exocytosis, the released CG protein functions to block the polyspermy by modifying the oocyte extracellular matrix, such as the zona pellucida [38]. Similar to our results, previous studies have reported that supplementation with GA and AG during the last 22 h of IVM has a positive effect on CG exocytosis [19]. However, further studies are needed to clarify the mechanism through which HA components, when added during oocyte IVM, exert a beneficial effect on CG exocytosis.

In our study, no expansion of cumulus cells was observed when GA and AG were added to the defined medium containing 0.1% PVA. Conversely, in the complex medium containing 10% PFF, cumulus expansion was significantly increased in the GA- and AG-treated COCs compared with the untreated COCs. To investigate the effect of GA and AG in complex media on the expression of genes related to cumulus cell expansion, RT-PCR was performed to analyze the mRNA expression levels of *CD44* or *CX43* in cumulus cells isolated from COCs matured in a complex culture medium with 10% PFF. *CD44* is the principal cell-surface receptor for the ECM glycosaminoglycan HA. *CD44* receptor synthesis is induced by gonadotropin during oocyte maturation [39,40]. Localization of *CD44,* a major cell-surface receptor for HA, in cumulus cells [41], suggests that the HA-*CD44* interaction may contribute to oocyte maturation. *CD44* expression in cumulus cells is a potential marker of oocyte capacity [42], and an increase in follicular fluid *CD44* is associated with high-quality oocytes [43]. In our study, *CD44* gene expression was significantly increased in cumulus cells treated with GA and AG compared with the control. Thus, these results indicate that the increased HA-*CD44* interaction following the addition of GA and AG enhances meiotic resumption during oocyte maturation.

GJCs are regions adjacent to the membranes of adjoining cells, which allow cells to exchange small molecules, thereby regulating their activity [34]. The HA-*CD44* interaction regulates the tyrosine phosphorylation of connexin 43 (a major gap junction protein found in COCs), closing gap junctions, and subsequently activating the activity of the maturation-promoting factor [44]. Interestingly, Wang et al. [45] found that *CX43* levels in human cumulus cells were positively correlated with embryo quality, based on cleavage rate and morphology, and were significantly higher in patients who were pregnant than in those who were not. Our results can also imply that the addition of GA and AG during cumulus cell expansion increased the HA-*CD44* interaction and increased the expression of *CX43* in cumulus cells, thereby improving the quality of oocytes.

Considering the importance of cumulus expansion, its study is necessary for evaluating IVM oocyte suitability for animal cloning and the production of transgenic animals. Thus, we demonstrated that high-quality oocytes matured in IVM medium supplemented with GA and AG presented high developmental competence when used as donor oocytes for SCNT. Therefore, it is expected that the addition of GA and AG may assist in the production of livestock when high-quality oocytes are produced in vitro.

## 5. Conclusions

In conclusion, the combined treatment of 0.05 mM GA and 0.05 mM AG during the initial 22 h of IVM improved the expression of genes related to cumulus expansion in cumulus cells or antioxidant-related genes in oocytes. In addition, the supplementation with GA and AG had beneficial effects on cumulus expansion, oocyte diameter, PVS thickness, ROS levels, CG exocytosis, and early embryonic development after PA and SCNT.

## Figures and Tables

**Table 1:** Primers used for gene expression analyses.

Gene	Primer Sequences (5′-3′)	Product Size (bp)	GenBank Accession Number
*GAPDH*	F: 5′-GTCGGTTGTGGATCTGACCT-3′	207	NM_001206359
	R: 5′-TTGACGAAGTGGTCGTTGAG-3′		
*RN18S*	F: 5′-CGCGGTTCTATTTTGTTGGT-3′	219	NR_046261.1
	R: 5′-AGTCGGCATCGTTTATGGTC-3′		
*CD44*	F: 5′-AGTCAAGAAGGTGAGGCAAA-3′	175	XM_021085286.1
	R: 5′-TGCCATTGTTAATCACCAGC-3′		
*CX43*	F: 5′-ACTGAGCCCCTCCAAAGACT-3′	191	NM_001244212
	R: 5′-GCTCGGCACTGTAATTAGCC-3′		
*GSR*	F: 5′-TGGGCTCTAAGACGTCACTG-3′	168	XM_003483635.4
	R: 5′-TCTATGCCAGCATTCTCCAG-3′		
*PRDX1*	F: 5′-CTTGATATCAGACCCCAAGC-3′	187	XM_021096742.1
	R: 5′-GAACTGGAAGGCCTGAACTA-3′		
*TXN2*	F: 5′-CAGGATGGACCTGACTTTCA-3′	192	NM_001243705.1
	R: 5′-GTACTCGATGGCGAGGTCT-3′		
*SOD1*	F: 5′-GTGCAGGGCACCATCTACTT-3′	222	NM_001190422.1
	R: 5′-AGTCACATTGCCCAGGTCTC-3′		

**Table 2:** Effect of glucuronic acid treatment during in vitro maturation (IVM) on embryonic development after parthenogenetic activation.

Glucuronic Acid Treatment (mM)	% of Oocytes That Reached MII	Number of Oocytes Cultured *	% of Embryos Developed to:		Number of Cells in Blastocyst
			≥2 Cells	Blastocyst	
Control	94.4 ± 1.1	133	93.6 ± 2.5	51.0 ± 4.5	31.5 ± 1.3 ^a^
0.01	94.7 ± 2.4	139	85.5 ± 6.4	45.3 ± 2.1	34.2 ± 1.5 ^ab^
0.05	91.6 ± 0.8	131	91.6 ± 3.2	42.0 ± 5.0	38.0 ± 2.0 ^b^
0.1	91.3 ± 2.1	134	88.2 ± 2.6	49.9 ± 3.4	34.8 ± 1.6 ^ab^

* Four replicates; ^a, b^ Values in the same column with different superscript letters are significantly different *(p* < 0.05); IVM media: M199 with 0.1% polyvinyl alcohol.

**Table 3:** Effect of N-acetyl-D-glucosamine treatment during in vitro maturation (IVM) on embryonic development after parthenogenetic activation.

N-Acetyl-D-Glucosamine Treatment (mM)	% of Oocytes That Reached MII	Number of Oocytes Cultured *	% of Embryos Developed to:		Number of Cells in Blastocyst
			≥2 Cells	Blastocyst	
Control	95.0 ± 1.6	137	85.0 ± 2.5 ^a^	46.3 ± 4.1 ^a^	41.8 ± 1.8
0.01	96.9 ± 1.2	149	91.1 ± 1.5 ^ab^	48.5 ± 5.8 ^ab^	43.1 ± 1.9
0.05	95.8 ± 1.7	148	91.8 ± 3.3 ^b^	59.6 ± 3.9 ^b^	40.0 ± 1.6
0.1	97.2 ± 1.6	149	89.6 ± 1.9 ^ab^	45.2 ± 3.2 ^a^	41.5 ± 1.8

* Four replicates; ^a, b^ Values in the same column with different superscript letters are significantly different (*p* < 0.05); IVM media: M199 with 0.1% polyvinyl alcohol.

**Table 4:** Effect of glucuronic acid (GA) and N-acetyl-D-glucosamine (AG) treatment during in vitro maturation (IVM) on embryonic development after parthenogenetic activation.

IVM Treatment ^A^	% of Oocytes That Reached MII	Number of Oocytes Cultured *	% of Embryos Developed to:		Number of Cells in Blastocyst
			≥2 Cells	Blastocyst	
Control	92.6 ± 0.3	175	94.5 ± 2.5	43.9 ± 2.9 ^a^	39.7 ± 1.8
GAAG0-22	92.8 ± 0.7	174	89.2 ± 2.8	53.6 ± 3.2 ^b^	39.6 ± 1.6
GAAG23-44	94.9 ± 1.7	173	89.7 ± 2.5	44.5 ± 0.7 ^a^	39.0 ± 1.8
GAAG0-44	92.5 ± 2.9	176	91.0 ± 1.7	49.2 ± 3.1 ^ab^	41.0 ± 1.5

* Four replicates; ^a, b^ Values in the same column with different superscript letters are significantly different (*p* < 0.05); ^A^ M199 with 0.1% polyvinyl alcohol.

**Table 5:** Effect of glucuronic acid (GA) and N-acetyl-D-glucosamine (AG) treatment during in vitro maturation (IVM) on oocyte diameter and size of the perivitelline space (PVS).

IVM Treatment ^A^	Number of Oocytes Tested *	Diameter of Oocyte (μm)	PVS Size (μm)
Control	60	110.0 ± 0.3 ^a^	4.35 ± 0.19 ^a^
GAAG0-22	60	111.5 ± 0.5 ^b^	5.08 ± 0.27 ^b^
GAAG23-44	60	110.4 ± 0.4 ^ab^	4.40 ± 0.26 ^a^
GAAG0-44	60	110.6 ± 0.5 ^ab^	4.47 ± 0.24 ^ab^

* Three replicates; ^a, b^ Values in the same column with different superscript letters are significantly different (*p* < 0.05); ^A^ M199 with 0.1% polyvinyl alcohol.

**Table 6:** Effect of glucuronic acid (GA) and N-acetyl-D-glucosamine (AG) treatment during in vitro maturation (IVM) on reactive oxygen species (ROS) level in matured oocyte and fluorescent intensities of cortical granules in oocyte after parthenogenetic activation (PA).

IVM Treatment ^A^	Number of Oocytes Tested *	Relative ROS Level (Pixels/Oocyte)	Number of Oocytes Tested *	Cortical Granules (Pixels/Oocyte)
Control	68	1.00 ± 0.02 ^a^	52	1.0 ± 0.02 ^a^
GAAG0-22	68	0.75 ± 0.03 ^b^	53	0.9 ± 0.02 ^b^
GAAG23-44	68	0.91 ± 0.03 ^c^	NA	
GAAG0-44	68	0.70 ± 0.03 ^b^	NA	

* Three replicates; ^a, b^ Values in the same column with different superscript letters are significantly different (*p* < 0.05); ^A^ M199 with 0.1% polyvinyl alcohol; NA: not applicable.

**Table 7:** Effect of glucuronic acid (GA) and N-acetyl-D-glucosamine (AG) treatment during in vitro maturation (IVM) on embryonic development after parthenogenetic activation.

IVM Treatment		% of Oocytes that Reached MII	Number of Oocytes Cultured *	% of Embryos Developed to:		Number of Cells in Blastocyst
				≥2 Cells	Blastocyst	
0.1% PVA ^A^	GAAG0-22	93.2 ± 0.7	178	89.8 ± 0.5	52.5 ± 3.3 ^a^	37.5 ± 1.8 ^a^
10% PFF ^B^	Control	92.4 ± 2.6	172	89.7 ± 4.1	53.0 ± 6.1 ^a^	45.4 ± 1.9 ^b^
	GAAG0-22	93.2 ± 0.7	180	92.7 ± 0.9	66.6 ± 4.0 ^b^	43.9 ± 1.7 ^b^

* Four replicates; ^a,b^ Values in the same column with different superscript letters are significantly different (*p* < 0.05); ^A^ M199 with 0.1% polyvinyl alcohol with 0.05 mM GA and 0.05 mM AG; ^B^ M199 with 10% porcine follicular fluid.

**Table 8:** Effect of glucuronic acid (GA) and N-acetyl-D-glucosamine (AG) treatment during in vitro maturation (IVM) on embryonic development after somatic cell nuclear transfer.

IVM Treatment ^A^	% of Oocytes That Reached MII	% of Oocytes Fused	Number of Oocytes Cultured *	% of Embryos Developed to:		Number of Cells in Blastocyst
				≥2 Cells	Blastocyst	
Control	88.5 ± 2.5	77.2 ± 10.3	125	80.9 ± 5.6	16.0 ± 1.6 ^a^	38.7 ± 4.2
GAAG0-22	92.1 ± 1.3	83.5 ± 4.6	146	84.7 ± 2.7	25.1 ± 1.8 ^b^	41.8 ± 3.1

* Four replicates; ^a,b^ Values in the same column with different superscript letters are significantly different (*p* < 0.05); ^A^ M199 with 10% porcine follicular fluid.

## Data Availability

The data presented in this study are available on request from the corresponding author.
